# Insights into foundational therapies for heart failure with reduced ejection fraction

**DOI:** 10.1002/clc.23847

**Published:** 2022-07-05

**Authors:** John J. V. McMurray, Kieran F. Docherty

**Affiliations:** ^1^ British Heart Foundation Glasgow Cardiovascular Research Centre University of Glasgow Glasgow UK

**Keywords:** Heart failure with reduced ejection fraction

## Abstract

In this review, we discuss what is meant by “foundational” therapy for patients with heart failure and reduced ejection fraction (HFrEF) and the evidence supporting the use of the five agents that comprise this group of drugs i.e., sacubitril/valsartan, a beta‐blocker, an aldosterone or mineralocorticoid receptor antagonist (MRA) and a sodium‐glucose cotransporter 2 (SGLT2) inhibitor. We review the conventional approach to sequencing these therapies in HFrEF and proposed new rapid sequencing strategies. We review a recent modelling study suggesting the optimal sequence of treatment includes a sodium‐glucose cotransporter 2 inhibition and an MRA as the first two therapies. Finally, we review the important opportunity offered by hospitalization for worsening heart failure to initiate and optimize foundational therapies in patients at high risk of early adverse outcomes.

## 1 INTRODUCTION

The term “foundational” therapy has been used to describe the key life‐saving pharmacological treatments which should form the bedrock or foundations of drug and device management of patients with heart failure and reduced ejection fraction (HFrEF). While several other pharmacological agents and devices, such as an implantable cardioverter‐defibrillator and cardiac resynchronization therapy, also have valuable benefits these are used as “second line” treatment, added to “foundational therapy” in selected patients. As such, “foundational” therapy is strongly recommended in international guidelines for all patients who can tolerate it.^1^, ^2^

**Figure 1:**
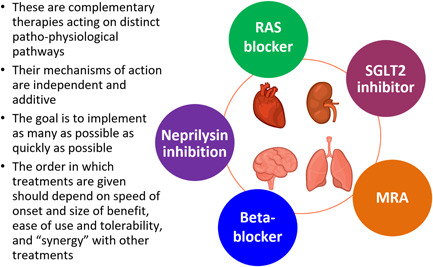
The “foundational” therapies for heart failure with a reduced ejection fraction (HFrEF). MRA, mineralocorticoid receptor antagonist; RAS, renin angiotensin system; SGLT2, sodium‐glucose cotransporter 2

Currently, “foundational” therapy consists of sacubitril/valsartan, a beta‐blocker, an aldosterone or mineralocorticoid receptor antagonist (MRA) and a sodium‐glucose cotransporter 2 (SGLT2) inhibitor.^3^, ^4^, ^5^, ^6^, ^7^, ^8^, ^9^, ^10^, ^11^, ^12^, ^13^ In other words, “foundational” therapy includes five distinct pharmacological interventions administered as four pills, as sacubitril/valsartan is the combination of a renin‐angiotensin system (RAS) blocker and a neprilysin inhibitor, given in a single preparation. The fundamentally important principle is that the efficacy of each is independent of the other and that the benefits of these treatments are additive (Figure 1). Consequently, when used together, these treatments are estimated to result in substantial cumulative lifetime gains in overall survival, and survival free of hospital admission for worsening heart failure.^14^

Until recently, guidelines recommended a gradual, stepwise, introduction of these therapies in the order in which the landmark clinical trials using each treatment were conducted. Moreover, it was recommended that the dose of each therapy was titrated to the target dose used in the relevant trial before the next treatment was added.^15^, ^16^ Practically, this meant starting with a RAS blocker i.e., an angiotensin‐converting enzyme (ACE) inhibitor or angiotensin receptor blocker (ARB), adding a beta‐blocker, then an MRA, followed by a neprilysin inhibitor (by switching from an ACE inhibitor or ARB to sacubitril valsartan) and, if this approach was continued, finally starting an SGLT2 inhibitor (the most recent “foundational” therapy). If the dose of each treatment was titrated to the target dose before initiating the next, the commencement of all “foundational” therapies could take as long as 6 months, with patients not receiving several of these life‐saving therapies for much of this period. This paradigm is now considered outdated and a more rapid introduction of “foundational” therapy has recently been advocated.^1^, ^2^, ^17^, ^18^ Crucially, the newly recommended strategy prioritizes the initiation of each therapy as quickly as possible over the uptitration of the dose of any individual therapy. While attempting to titrate to the target dose is still essential, it is now considered to be a secondary goal, with starting all four life‐saving “foundational” treatments, at least in a low dose, as the primary objective. This is because, as discussed below, even sub‐target doses of these therapies have substantial benefits which are apparent within weeks of starting treatment. Also, the new guidelines no longer recommend initiating treatment in the historical sequence, following the chronology of the clinical trials, and the order in which each foundational therapy is started does not matter and may be based on other considerations such as potential pharmacological synergies related to safety and patient profile and likely tolerability. In the remainder of this article, we will explain the basis of this paradigm shift in our approach to the pharmacological management of HFrEF.

### 1.1 “Foundational” therapy for HFrEF

Central to the concept of a “foundational” therapy, strongly recommended for every patient who can tolerate it, is the necessity for irrefutable evidence of a clinically meaningful improvement in survival. For all of the 4 “foundational” treatments for HFrEF, such evidence is available from large, randomized, placebo or active‐controlled trials where each therapy has been shown to reduce the risk of cardiovascular (and all‐cause) mortality, along with the risk of hospitalization for worsening heart failure.^1^, ^2^, ^3^, ^4^, ^5^, ^6^, ^7^, ^8^, ^9^, ^10^, ^11^, ^12^, ^13^ Indeed, these benefits have been demonstrated in at least two trials for all “foundational” treatments except sacubitril‐valsartan where the statistical significance in the single large trial exceeded the equivalent of two individual trials (*p* < .00125). Furthermore, most of these “foundational” treatments have similar benefits in reducing mortality in patients with left ventricular systolic dysfunction, acute heart failure, or both at the time of acute myocardial infarction.^19^, ^20^, ^21^, ^22^, ^23^, ^24^, ^25^

It is important to reiterate that other pharmacological treatments including digoxin, ivabradine, and vericiguat have worthwhile benefits and may be useful second‐line therapies in selected patients.^1^, ^2^ These treatments do not have as strong a recommendation because they have not been shown to reduce mortality and do have as robust an evidence base as the “foundational” therapies. The combination of hydralazine and isosorbide dinitrate is the one other pharmacological approach that has a strong recommendation for patients self‐identifying as African American, although the evidence‐base for this therapeutic combination is not robust.^1^, ^2^

### 1.2 Conventional sequencing of “foundational” therapies for HFrEF

As described above, prior international guidelines on the management of HFrEF recommended a stepwise introduction of treatment the order of which was not based on any evidence other than the chronology of the landmark evidence‐generating trials.^17^, ^18^ Implicit in the former recommendations was the assumption that the treatments studied earlier were either more efficacious or better tolerated than those identified more recently i.e., this would be a justification for prioritization their initiation. Clearly, this is not correct and the SGLT2 inhibitors, in particular, have demonstrated remarkable tolerability, as well as efficacy, compared to RAS blockers (even though they were added to RAS blockers).^12^, ^13^ The historical sequencing approach also implies that the benefit of a beta‐blocker is dependent on background treatment (at the target dose) with an ACE inhibitor, the benefits of an MRA are dependent on background treatment with both an ACE inhibitor and beta‐blocker and so on. We know this is not the case. For example, in CONSENSUS, more than half of the patients were on background MRA therapy and in RALES only 10% of patients were taking a beta‐blocker at baseline.^3^, ^9^ In addition, trials of new treatments did not require all patients to be taking the target dose of prior proven therapies. All the evidence we have shows that “foundational” therapies act through distinct pharmacological mechanisms and that their effects are independent and additive and that there is no pharmacological rationale for choosing to initiate one “foundational” therapy in preference to another i.e., any of these treatments can be used first.^17^, ^18^, ^26^, ^27^, ^28^, ^29^

The former recommendation to uptitrate each therapy to the target dose (or maximally tolerated dose below that) before adding the next has also changed, for two reasons. First, the recent SGLT2 inhibitor trials demonstrated a very early benefit of therapy (within a month of starting treatment). Although highlighted by the recent SGLT2 inhibitor trials, this finding was also true for most other foundational therapies.^30^, ^31^, ^32^, ^33^, ^34^, ^35^ Therefore, the gradual, sequential introduction of therapies, with dose uptitration before the next step, delays the introduction of other life‐saving treatments. Second, even the sub‐target doses of most “foundational” therapies used during the uptitration phase in the landmark trials had notable (and, by definition, early) benefits.^30^, ^31^, ^32^, ^33^, ^34^, ^35^ Collectively, these observations argue for starting as many “foundational” therapies as possible as quickly as possible, recognizing that uptitration to the target dose will have to come later (and should not be forgotten and has additional benefits).^36^, ^37^ We believe that this emphasis on speed will minimize unnecessary deaths and hospitalizations in patients with HFrEF. Indeed, the previously recommended approach to treatment was both time‐consuming and labor‐intensive, requiring multiple clinic visits. This may have, in part, explained why relatively few patients with HFrEF received all 4 “foundational” therapies and rarely at the target dose.^38^, ^39^

## 2 PROPOSED NEW RAPID SEQUENCING STRATEGIES FOR PATIENTS WITH HFREF

New international guidelines support the rapid initiation of “foundational” therapies and novel sequencing strategies have been proposed, one of which advocated establishing patients on all 4 “foundational” treatments within 4 weeks.^17^, ^18^, ^40^, ^41^ These proposals are based on the rationale described above and also on the ability of one treatment to enhance the tolerability and safety of another e.g. SGLT2 inhibitors and sacubitril/valsartan reduce the risk of hyperkalemia in patients taking an MRA. In reviews of this paradigm shift in approach to starting therapy and in the new guidelines, the possibility of even starting two treatments simultaneously has been proposed (although this will not always be possible).^17^, ^18^, ^40^, ^41^

### 2.1 New sequences based on hypothetical pharmacological synergies

Using this rationale, one strategy suggested is, as a first step, initiation of a low dose of an evidence‐based beta‐blocker (bisoprolol, metoprolol succinate or carvedilol) along with an SGLT2 inhibitor (dapagliflozin or empagliflozin) in patients who are assessed to be clinically euvolemic. Beta‐blockers are an attractive first step as they lead to a large reduction in death, including sudden death.^7^, ^32^ The CIBIS III trial showed that starting treatment with a beta‐blocker, compared with an ACE inhibitor, was non‐inferior in terms of efficacy.^42^ However, there is a small risk of early worsening of heart failure caused by beta‐blockers and the short‐term diuretic effect of SGLT2 inhibitors may help offset this.^43^, ^44^ Establishing the renoprotective benefits of SGLT2 inhibitors before starting a renin‐angiotensin blocker and MRA, which can lead to deterioration in kidney function, is also hypothetically attractive.^13^, ^45^ Step 2 of this strategy involves initiation of sacubitril/valsartan, 1–2 weeks later. This does not delay adding a neprilysin inhibitor to a renin‐angiotensin blocker as in prior guidelines (which initiated an ACE inhibitor ARB and later switched to sacubitril/valsartan^15^, ^16^). Combined neprilysin inhibitor/renin‐angiotensin blockade reduced death from boh worsening heart failure and sudden cardiac death and heart failure hospitalization compared with a renin‐angiotensin blocker alone.^11^, ^46^ Furthermore, the addition of a neprilysin inhibitor reduces the rate of decline in kidney function over time and the risk of hyperkalemia with MRAs, benefits shared with SGLT2 inhibitors.^47^, ^48^ Pretreatment with an SGLT2 inhibitor and sacubitril/valsartan may, therefore, increase the likelihood of safely introducing and maintaining a patient on MRA therapy subsequently.^11^, ^46^, ^47^, ^48^, ^49^, ^50^, ^51^, ^52^ The third step of this proposal is the introduction of an MRA after an additional 1–2 weeks, kidney function and potassium permitting i.e., eGFR ≥30 ml/min/1.73 m^2^ and potassium ≤5.0 mmol/L. After these three steps, an attempt should be made to uptitrate the doses of all four “foundational” to the targets used in the randomized clinical trials. It is important to note, however, that many patients in those trials did not reach the target and the maximally tolerated dose below the target is acceptable and consistent with the evidence‐based strategy employed in the landmark trials (this exception is SGLT2 inhibitors as these are used in a single dose and do not require titration).^35^

### 2.2 New sequences based on the clinical profile of patients

Alternative, but not mutually exclusive, approaches emphasize the selection of initial therapy according to the patient's clinical profile e.g., use of sacubitril/valsartan earlier in hypertensive individuals.^53^, ^54^, ^55^

### 2.3 New sequences based on mathematical modeling

Recently, using data from trials in HFrEF, Shen and colleagues modeled the potential reductions in events that might result from (i) more rapid introduction and uptitration of the “foundational” therapies used in the historical order recommended in previous guidelines and (ii) more rapid introduction and uptitration combined with the use of these treatments in a variety of different sequences.^56^ Over the first 12 months of therapy, simply accelerating the introduction and uptitration of therapy led to 23 fewer patients per 1000 treated experiencing the composite of heart failure hospitalization or cardiovascular death and 7 fewer deaths from any cause. The optimal alternative sequences of treatment always included SGLT2 inhibition and an MRA as the first two therapies. Others have supported an “SGLT2 inhibitor first” strategy.^57^

### 2.4 The opportunity presented by hospitalization

Patients admitted to the hospital with heart failure represent a group at particularly high risk over the coming weeks and months.^1^, ^2^, ^58^ To maximize protection during the post‐discharge “vulnerable” period, all “foundational” therapies for HFrEF should be started in‐hospital if at all possible.^58^ The importance of this approach is emphasized by the evidence that deferring treatment initiation until after discharge results in lower rates of use.^59^ In‐hospital initiation of “foundational” therapies is strongly endorsed in the new international guidelines.^1^, ^2^

## 3 CONCLUSION

There is unequivocal evidence that sacubitril/valsartan, a beta‐blocker, an MRA and an SGLT2 inhibitor are the “foundational” therapies that should, where possible, be prescribed to all patients with HFrEF to maximize their survival and minimize their risk of worsening heart failure, leading to deterioration in symptoms and quality of life and admission to hospital. The benefits of these 4 treatments are obtained quickly after initiation and are additive. Rapid and safe implementation of these 4 foundational therapies is feasible and now a therapeutic imperative.
